# Indoxyl sulfate mediates low handgrip strength and is predictive of high hospitalization rates in patients with end-stage renal disease

**DOI:** 10.3389/fmed.2023.1023383

**Published:** 2023-02-02

**Authors:** Yi-Chou Hou, Yueh-Min Liu, Min-Ter Liao, Cai-Mei Zheng, Chien-Lin Lu, Wen-Chih Liu, Kuo-Chin Hung, Shyh-Min Lin, Kuo-Cheng Lu

**Affiliations:** ^1^Division of Nephrology, Department of Internal Medicine, Cardinal Tien Hospital, New Taipei City, Taiwan; ^2^School of Medicine, Fu Jen Catholic University, New Taipei City, Taiwan; ^3^Department of Nursing, Ching Kuo Institute of Management and Health, Keelung, Taiwan; ^4^Department of Pediatrics, Taoyuan Armed Forces General Hospital, Hsinchu, Taiwan; ^5^Department of Pediatrics, Tri-Service General Hospital, National Defense Medical Center, Taipei City, Taiwan; ^6^Division of Nephrology, Department of Internal Medicine, Taipei Medical University-Shuang Ho Hospital, New Taipei City, Taiwan; ^7^Division of Nephrology, Department of Internal Medicine, School of Medicine, College of Medicine, Taipei Medical University, Taipei City, Taiwan; ^8^Taipei Medical University-Research Center of Urology and Kidney (TMU-RCUK), School of Medicine, College of Medicine, Taipei Medical University, Taipei City, Taiwan; ^9^Division of Nephrology, Department of Medicine, Fu Jen Catholic University Hospital, School of Medicine, Fu Jen Catholic University, New Taipei City, Taiwan; ^10^Division of Nephrology, Department of Internal Medicine, Taipei Hospital, Ministry of Health and Welfare, New Taipei City, Taiwan; ^11^Department of Biology and Anatomy, National Defense Medical Center, Taipei City, Taiwan; ^12^Division of Nephrology, Department of Medicine, Min-Sheng General Hospital, Taoyuan City, Taiwan; ^13^Division of Radiology, Department of Medicine, Cardinal Tien Hospital, New Taipei City, Taiwan; ^14^Division of Nephrology, Department of Medicine, Taipei Tzu Chi Hospital, Buddhist Tzu Chi Medical Foundation, New Taipei City, Taiwan

**Keywords:** indoxyl sulfate, irisin, chronic kidney disease, sarcopenia, handgrip strength, frailty

## Abstract

**Background and aims:**

Sarcopenia has a higher occurrence rate in patients with chronic kidney disease (CKD) and end-stage renal disease (ESRD) than in the general population. Low handgrip strength—and not sarcopenia *per se*—is associated with clinical outcomes in patients with CKD, including cardiovascular mortality and hospitalization. The factors contributing to low handgrip strength are still unknown. Accordingly, this study aimed to determine whether uremic toxins influence low handgrip strength in patients with CKD.

**Materials and methods:**

This cohort study lasted from August 2018 to January 2020. The participants were divided into three groups: the control group [estimated glomerular filtration rate (eGFR) ≥ 60 ml/min], an advanced CKD group (eGFR = 15–60 ml/min), and an ESRD group (under maintenance renal replacement therapy). All participants underwent handgrip strength measurement, dual-energy X-ray absorptiometry, and blood sampling for myokines (irisin, myostatin, and interleukin 6) and indoxyl sulfate. Sarcopenia was defined according to the Asian Working Group for Sarcopenia consensus as low appendicular skeletal muscle index (appendicular skeletal muscle/height^2^ of < 7.0 kg/m^2^ in men and < 5.4 kg/m^2^ in women) and low handgrip strength (< 28 kg in men and < 18 kg in women).

**Results:**

Among the study participants (control: *n* = 16; CKD: *n* = 17; and ESRD: *n* = 42), the ESRD group had the highest prevalence of low handgrip strength (41.6 vs. 25% and 5.85% in the control and CKD groups, respectively; *p* < 0.05). The sarcopenia rate was similar among the groups (12.5, 17.6, and 19.5% for the control, CKD, and ESRD groups, respectively; *p* = 0.864). Low handgrip strength was associated with high hospitalization rates within the total study population during the 600-day follow-up period (*p* = 0.02). The predictions for cardiovascular mortality and hospitalization were similar among patients with and without sarcopenia (*p* = 0.190 and *p* = 0.094). The serum concentrations of indoxyl sulfate were higher in the ESRD group (227.29 ± 92.65 μM vs. 41.97 ± 43.96 μM and 6.54 ± 3.45 μM for the CKD and control groups, respectively; *p* < 0.05). Myokine concentrations were similar among groups. Indoxyl sulfate was associated with low handgrip strength in univariate and multivariate logistic regression models [univariate odds ratio (OR): 3.485, 95% confidence interval (CI): 1.372–8.852, *p* = 0.001; multivariate OR: 8.525, 95% CI: 1.807–40.207, *p* = 0.007].

**Conclusion:**

Handgrip strength was lower in the patients with ESRD, and low handgrip strength was predictive of hospitalization in the total study population. Indoxyl sulfate contributed to low handgrip strength and counteracted the benefits of myokines in patients with CKD.

## 1. Introduction

Chronic kidney disease (CKD) is defined as impaired glomerular filtration caused by structural or chronic damage to the glomerulus and the genitourinary tract. The risk factors for CKD include advanced age, metabolic disorders such as diabetes mellitus, uncontrolled hypertension, autoimmune disorders such as systemic lupus erythematous, and hereditary disorders such as polycystic kidney disease (1). The progressive decrease in glomerular filtration rate (GFR) can result in multiple complications, such as the activation of the renin–angiotensin–aldosterone system, insulin resistance, secondary hyperparathyroidism, vitamin D deficiency, electrolyte imbalance, and the accumulation of uremic toxins, which can trigger further comorbidities (2–4). Patients with CKD and end-stage renal disease (ESRD) are more vulnerable to comorbidities such as uncontrolled congestive heart failure, fluid overload, altered consciousness, decreased erythropoiesis, renal osteodystrophy, sarcopenia, and frailty; therefore, these patients have a higher incidence of hospitalization or mortality than the general population (5–8).

Frailty is defined as the state of increased vulnerability resulting from aging-associated decline with compromised coping ability for daily or acute stressors due to loss in reserve and function across multiple physiologic systems (9). Sarcopenia, a major component of frailty, is defined as age-related loss of muscle mass, plus low muscle strength, and/or low physical performance. (10). The intrinsic contraction–extension pattern of skeletal muscle supports the posture and structure of the body. Sarcopenia is diagnosed either through physical assessments, such as a handgrip strength test, or by using radiology tools such as dual-energy X-ray absorptiometry (DEXA) (11). Myokines such as irisin or myostatin are released from the skeletal muscle, and they play a role in the modulation of skeletal muscle homeostasis and by extension the development of sarcopenia and frailty (12). Patients with CKD are more susceptible to frailty or sarcopenia due to advanced age with other comorbidities such as insulin resistance or metabolic acidosis, and dysregulation of anabolic myokines (13, 14). Sarcopenia diagnosed through physical assessment, as opposed to radiology, was associated with poorer clinical outcomes in patients with CKD. However, the mechanisms behind this relationship are still under investigation (15).

Indoxyl sulfate is a protein-bound uremic toxin found in patients with CKD. The accumulation of this uremic toxin could directly increase the oxidative stress levels within skeletal muscle cells and impair the function of mitochondria (16). Because skeletal muscle is part of the cardiovascular system, this could affect catabolism and accelerate the development of frailty and sarcopenia (17, 6). Furthermore, indoxyl sulfate influenced decrease skeletal muscle anabolism, resulting in atrophy (17). For patients undergoing dialysis, handgrip strength is related to frailty, and in patients with CKD, handgrip strength is related to specific comorbidities or mortality (18, 19). However, few clinical studies have researched the relationship of uremic toxins with sarcopenia and frailty or the interaction between myokines and body composition. Accordingly, the present study investigated the role of myokines in contributing to low handgrip strength in patients with CKD and ESRD.

## 2. Materials and methods

### 2.1. Ethics and study protocol

This study was conducted at a regional hospital in New Taipei City, Taiwan, in accordance with the tenets of the Declaration of Helsinki. The study protocol was approved by the Ethics Committee of Human Studies at Cardinal Tien Hospital (CTH-107-3-5-027). The study period lasted from August 2018 to January 2020. The inclusion criteria were as follows: (a) estimated GFR (eGFR) < 60 ml/min or spot urine proteinuria > 200 mg/g, (b) age > 20 years, and (c) able to communicate verbally in Mandarin Chinese. The exclusion criteria were as follows: having unstable angina, acute myocardial infarction during the past 6 months, severe anemia (Hb < 8 g/dL), systolic hypertension (> 190 mmHg), active inflammation or infection, malignant cancer, autoimmune diseases, emotional instability, musculoskeletal disability, uncontrolled cardiac failure or respiratory problems, or being hospitalized during the past month. After the participants were enrolled, they were divided into three groups: (1) the control group (eGFR > 60 ml/min), (2) the CKD group (eGFR 15–60 ml/min), and (3) the ESRD group. ESRD was defined as receiving maintenance renal replacement therapy continuously for > 3 months. For the ESRD patients, the participants received hemodialysis (three times per week with duration 3–4 h for each session) with polyethersulfone as the dialyzer materials. The Kt/V of all participants was higher than 1.2 as the suggestion of the National Kidney Foundation’s Kidney Disease Outcomes Quality Initiative (20).

All participants received a prestudy medical workup, including a physical evaluation, electrocardiography, resting echocardiography, and blood biochemical tests. After enrollment, the participants received assessments of their clinical parameters, baseline hematological and biochemical parameters, baseline DEXA measurements, and myokine concentrations.

### 2.2. Demographic data and biochemical results

Demographic data were obtained from the medical records at Cardinal Tien Hospital. The diagnoses of congestive heart failure, diabetes mellitus, and hypertension were verified through medical records. Body weight and height were measured after hemodialysis to obtain body mass index. The following predialytic hematological and biochemical parameters were collected from each patient within 1 month after obtaining written informed consent: hemoglobin, platelet count, white blood cell count, glutamic oxaloacetic transaminase, glutamic pyruvic transaminase, albumin, blood sugar, uric acid, total cholesterol, triglycerides, sodium, potassium, calcium, phosphorus, and intact parathyroid hormone. The eGFR was determined by using the Modification of Diet in Renal Disease Study equation (21).

### 2.3. Measurement of myokines and indoxyl sulfate

Myokines, including irisin, myostatin, and interleukin-6, were measured by enzyme immunoassay kit (Abbkine, Wuhan, China). Serum samples were drawn to measure biochemical and hematological parameters. Serum was collected under fasting conditions and stored at −80°C for later measurement. The parameters were measured according to the manufacturer’s instructions (the inter- and intra-assay coefficients of variability for irisin, myostatin, and interleukin-6 were < 11% and < 9%, respectively). Indoxyl sulfate was measured using an enzyme-linked immunosorbent assay (ELISA) kit (Leadgene Biomedical, Tainan, Taiwan), validated through high-performance liquid chromatography–mass spectrometry (US patent: US10723791B2). The monoclonal antibody against antigenic indoxyl sulfate is generated from 8 to 10 week-old female BALB/c mice with removal rate 97.78% of human plasma. The binding activity, against indole, L-tryptophan or 3-indoleacetic acid (defined by mean absorbance of compounds–spiked wells)/[mean absorbance of blank control wells (B/Bo)] was less than 30%. Briefly, the serum was diluted by 20-fold to a final volume of 100 μL and then added to an equal volume of diluted detection antibody. After 1 h, the ELISA wells were washed, and 3,3’,5,5’-tetramethylbenzidine was used for color development. The indoxyl sulfate level was determined on the basis of a standard curve.

### 2.4. DEXA: measurement of muscle mass

In addition to blood sampling, DEXA imaging was also performed. Patients with ESRD received DEXA scans on the day after hemodialysis. The DEXA imaging process was as follows. The patients were asked to fast overnight and refrain from drinking alcohol for > 8 h before the DEXA scan. During the examination, the patients were asked to wear cotton clothing and remove all metal objects from their persons. Scans were performed using a GE Lunar iDXA (GE Healthcare, Chicago, IL, USA) operated in whole-body scan mode, and the scan was performed in the order of head, upper limbs, lower limbs, and trunk. The whole-body scan of each patient required approximately 20 min to complete.

The appendicular skeletal mass index was defined as the sum of the lean muscle mass of all four limbs divided by the patient’s height (in meters) squared (appendicular skeletal muscle/height^2^) (22). The relative fat mass indices for the trunk, leg, arm, android, gynoid, and total body fat were obtained according to the method described by Stults-Kolehmainen et al. (23). The scanner manufacturer defined the trunk, leg, android, and gynoid regions as follows: (1) The trunk region comprises the neck, chest, abdominal, and pelvic areas. The upper and lower perimeters of the trunk are the interior edge of the chin and the middle of the femoral necks without touching the brim of the pelvis, respectively. (2) The leg region comprises the pelvic region at an angle perpendicular to the femoral neck. (3) The android region comprises the area between the ribs and the top of the pelvis that is totally enclosed by the trunk region. The upper boundary is 20% of the distance between the iliac crest and the neck, and the lower boundary is at the top of the pelvis. (4) The gynoid region comprises the hips and upper thighs overlapping both the leg and trunk regions (24). The results of these scans were analyzed using the DEXA scanner’s integrated software (v12.10.017, GE Healthcare, Chicago, IL, USA).

### 2.5. Diagnoses of sarcopenia and low handgrip strength

The Asian Working Group for Sarcopenia (AWGS) (25) defines sarcopenia as low muscle mass and low muscle strength. We used the AWGS algorithm to identify sarcopenia as follows: appendicular skeletal muscle index (appendicular skeletal muscle/height^2^) of < 7.0 kg/m^2^ in men and < 5.4 kg/m^2^ in women. Low handgrip strength was defined a handgrip strength of < 28 kg in men and < 18 kg in women (25).

### 2.6. Cardiovascular mortality and hospitalization assessment

Cardiovascular mortality and hospitalization records were made prospectively by examining all patients who had been enrolled in the study for at least 3 months between 1 April 2018 and 31 December 2021. Each medical chart was reviewed, and a physician assigned the cause of death on the basis of all clinical information available from the Cardinal Tien Hospital emergency department or intensive care unit. Patients who were lost to follow-up after study completion were excluded from this analysis. Cardiovascular mortality was defined as any death directly related to cardiovascular system dysfunction occurring at Cardinal Tien Hospital (including stroke, myocardial infarction, congestive heart failure, or sudden death). The hospitalization assessment comprised all hospital stays lasting at least 1 night that occurred during the 2-year period after diagnosis. These data were collected from hospital admissions records and discharge letters extracted from the general practice records.

### 2.7. Statistics

Continuous variables are presented as mean ± standard deviation. Categorical values are expressed as percentages. A one-way analysis of variance was used to compare the differences in variables within the three patient groups. We used Pearson’s correlation coefficient to assess the predictive performance of individual parameters for low handgrip strength, including advanced age, diabetes mellitus, coronary artery disease, congestive heart failure, and indoxyl sulfate and myokine concentrations. Receiver operating characteristic (ROC) curves were plotted, and the area under the curve (AUC) was estimated. We compared the Kaplan–Meier estimates for 2-year cardiovascular mortality and hospitalization between the groups with and without sarcopenia and between the groups with and without low handgrip strength. All statistical analyses were performed using the statistical package SPSS for Windows (v.17; SPSS, Chicago, IL, USA). A two-tailed *p*-value of < 0.05 was considered statistically significant.

## 3. Results

### 3.1. Low handgrip strength was more prevalent than sarcopenia in patients with CKD and ESRD

Table 1 reveals the demographic characteristics of the three study groups. The numbers of participants in the control, CKD, and ESRD groups were 16, 17, and 42, respectively. The mean age of the participants in the control group (48.5 ± 10.94 years) was lower than that of the participants in the CKD and ESRD groups (65.64 ± 5.93 and 60.68 ± 14.81 years, respectively; *p* < 0.05). The rates of hypertension (18.75, 82.35, and 85.71%; *p* < 0.05) and diabetes mellitus (6.25, 64.7, and 64.9%, *p* < 0.05) were lower in the control group than in the CKD and ESRD groups. The percentage of female was higher in the control group (75, vs. 23.5% for CKD and 34.1% for ESRD group). The mean duration of the ESRD subjects receiving maintenance renal replacement therapy was 2.23 ± 1.45 years (not demonstrated in Table 1).

**TABLE 1:** Patient demographics by group.

	Control	CKD	ESRD	*p*-value
Sample size	16	17	41	
Age*	48.5 ± 10.94	65.64 ± 5.93	60.68 ± 14.81	*p* < 0.05
Female (%)	12 (75)	4 (23.5)	14 (34.1)	*P* < 0.05
Diabetes mellitus (%)*	1 (6.25)	11 (64.7)	26 (64.9)	*p* < 0.05
Hypertension (%)*	3 (18.75)	14 (82.35)	36 (85.71)	*p* < 0.05
Coronary artery disease (%)	0 (0)	1 (5.89)	4 (9.52)	*p* = 0.425
Congestive heart failure (%)^&^	0 (0)	1 (5.89)	17 (40.47)	*p* < 0.05
Malignancy (%)	0 (0)	1 (5.89)	1 (2.23)	*p* = 0.569

*Control vs. CKD; ^&^Control vs. ESRD.

Table 2 reports the prevalence of low handgrip strength and sarcopenia in the different groups. Low handgrip strength was more prevalent in the ESRD group than in the control and CKD groups (41.46 vs. 25% and 5.85%, respectively; *p* < 0.05). The prevalence of sarcopenia was similar among the groups (12.5, 17.6, and 19.5% for the control, CKD, and ESRD groups, respectively; *p* = 0.864). The body mass index was similar between groups (*p* = 0.398).

**TABLE 2:** Incidence of low handgrip strength and sarcopenia by group.

	Control	CKD	ESRD	*p*-value
Sample size	16	17	41	
Body mass index [kg/height (meter)^2^]	25.95 ± 4.79	24.12 ± 6.68	25.73 ± 3.44	*P* = 0.398
Grasping power (kg)^&^^	28.95 ± 9.64	31.63 ± 8.47	21.30 ± 9.43	*p* < 0.05
Case number of low handgrip strength (percentage)	4 (25)	1 (5.85)	17 (41.46)	*p* < 0.05
Case number of sarcopenia based on AWGS algorithm (percentage)	2 (12.5)	3 (17.6)	8 (19.5)	*P* = 0.804

*Control vs. CKD; ^&^Control vs. ESRD; ^CKD vs. ESRD.

Table 3 displays the results of hematological and biochemical analyses. The ESRD group exhibited significant baseline differences in blood urea nitrogen, creatinine, and eGFR when compared with the CKD and control groups. Moreover, the concentrations of sodium, albumin, and hemoglobin were significantly lower in the ESRD group than in the other two groups [sodium: 138.10 ± 2.54, 140.13 ± 2.39, and 140.73 ± 3.80 mEq/L (*p* < 0.05); albumin: 4.03 ± 0.34, 4.33 ± 0.42, and 4.23 ± 0.32 g/dL (*p* < 0.05); and hemoglobin: 10.95 ± 1.21, 13.17 ± 1.39, and 11.72 ± 2.19 g/dL, (*p* < 0.05) in the ESRD, control, and CKD groups, respectively]. Conversely, the concentrations of phosphorus was significantly higher in the ESRD group than in the other two groups [phosphorus: 5.44 ± 1.81, 3.81 ± 0.51, and 4.22 ± 0.72 mg/dL (*p* < 0.05); intact parathyroid hormone: 345.88 ± 291.67, 53.75 ± 25.82, and 157.43 ± 162.71 pg/ml (*p* < 0.05) in the ESRD, control, and CKD groups, respectively]. Serum indoxyl sulfate was significantly lower in the control group than in the other two groups (6.54 ± 3.45, 41.97 ± 43.96, and 227.29 ± 92.65 μM in the control, CKD, and ESRD groups, respectively; *p* < 0.05). Irisin was higher in the control group than in the other two groups, but the difference was non-significant. The concentrations of myostatin and interleukin-6 were similar among the groups.

**TABLE 3:** Hematological and biochemical results by group.

	Control	CKD	ESRD	*p*-value
Blood urea nitrogen (mg/dL)*^&^^	16.93 ± 9.25	44.91 ± 26.85	65.59 ± 21.44	*p* < 0.05*
Creatinine (mg/dL)*^&^^	0.76 ± 0.22	3.13 ± 2.06	9.70 ± 3.31	*p* < 0.05*
Estimated glomerular filtration rate (ml/min)*^&^^	97.77 ± 17.64	35.38 ± 25.75	7.45 ± 11.12	*p* < 0.05*
Sodium (mEq/L)^&^^	140.13 ± 2.39	140.73 ± 3.80	138.10 ± 2.54	*p* < 0.05*
Potassium (mEq/L)	4.17 ± 0.29	4.61 ± 0.62	4.35 ± 0.73	*p* = 0.156
Calcium (mg/dL)	9.61 ± 0.62	9.11 ± 0.70	9.09 ± 0.81	*p* = 0.081
Phosphorus (mg/dL)^&^^	3.81 ± 0.51	4.22 ± 0.72	5.44 ± 1.81	*p* < 0.05*
Uric acid (mg/dL)	5.71 ± 1.33	6.99 ± 1.58	6.35 ± 1.75	*p* = 0.115
Alkaline phosphatase (mg/dL)	68.87 ± 18.52	74.33 ± 28.77	80.53 ± 28.18	*p* = 0.313
Albumin (g/dL)^&^	4.33 ± 0.42	4.23 ± 0.32	4.03 ± 0.34	*p* < 0.05*
Cholesterol (mg/dL)	170.20 ± 27.78	143.73 ± 41.09	152.33 ± 39.61	*p* = 0.148
Triglyceride (mg/dL)	135.18 ± 54.59	118.82 ± 53.00	142.97 ± 69.62	*p* = 0.418
HbA1c (%)	5.86 ± 0.50	6.17 ± 0.74	6.58 ± 1.19	*p* = 0.138
Hemoglobin (g/dL)*^&^	13.17 ± 1.39	11.72 ± 2.19	10.95 ± 1.21	*p* < 0.05*
Indoxyl sulfate (μM)^&^^	6.54 ± 3.45	41.97 ± 43.96	227.29 ± 92.65	*p* ≤ 0.05*
TIrisin (pg/ml)	115.12 ± 108.73	73.13 ± 37.71	72.71 ± 59.61	*p* = 0.106
Myostatin (ng/ml)	2.64 ± 4.49	1.28 ± 0.84	1.29 ± 1.98	*p* = 0.188
Interleukin 6 (pg/ml)	3.67 ± 7.09	2.99 ± 5.09	4.68 ± 4.67	*p* = 0.232

*Control vs. CKD; ^&^Control vs. ESRD; ^CKD vs. ESRD.

Table 4 illustrated the concentration of myokine and indoxyl sulfate between the participants with and without lower handgrip strength. The indoxyl sulfate was higher in the lower handgrip strength group (172.06 ± 114.33 μM vs. 97.69 ± 121.93 μM, *p* < 0.05). The irisin concentration was lower in the lower handgrip strength group (68.86 ± 44.21 pg/ml, vs. 101.95 ± 88.77 pg/ml, *p* < 0.05).

**TABLE 4:** The concentration of myokine between the participants with and without lower handgrip strength.

Groups	With lower handgrip strength (*n* = 22)	Without lower handgrip strength (*n* = 52)	*P*-value
Female (percentage)	8 (36.3)	22 (42.2)	
Indoxyl sulfate (μM)	172.06 ± 114.33	97.69 ± 121.93	*P* < 0.05
Irisin (pg/ml)	68.86 ± 44.21	101.95 ± 88.77	*P* < 0.95
Myostatin (ng/ml)	1.28 ± 1.54	2.26 ± 3.56	*P* = 0.145
Interleukin 6 (pg/ml)	3.86 ± 4.89	4.33 ± 6.49	*P* = 0.302

### 3.2. Low handgrip strength—but not sarcopenia—was associated with hospitalization in all participants

**FIGURE 1:**
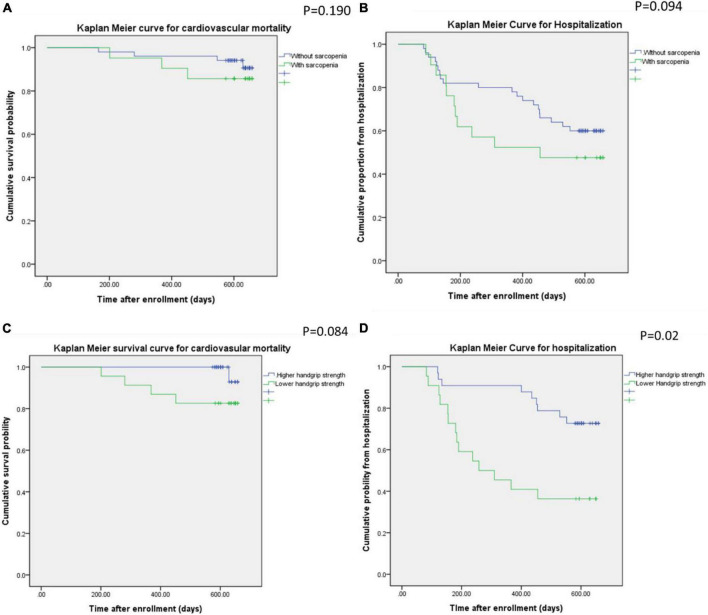
Kaplan–Meier curves for cardiovascular mortality and hospitalization in the participants with or without sarcopenia **(A,B)** and low handgrip strength **(C,B)**.

Figure 1 illustrates the Kaplan–Meier plot for cardiovascular mortality and hospitalization for all participants during the 2-year follow-up period, with stratification for sarcopenia and low handgrip strength. The differences in cardiovascular mortality and hospitalization rates between the participants with and without sarcopenia were non-significant (*p* = 0.191 for cardiovascular mortality; *p* = 0.094 for hospitalization). Conversely, the participants with low handgrip strength experienced significantly more hospitalizations (*p* = 0.02) during the 2-year follow-up period than the patients with normal handgrip strength did. Nevertheless, cardiovascular mortality rates were similar in the participants with and without low handgrip strength (*p* = 0.084).

### 3.3. Indoxyl sulfate was associated with low handgrip strength and clinical outcomes

**FIGURE 2:**
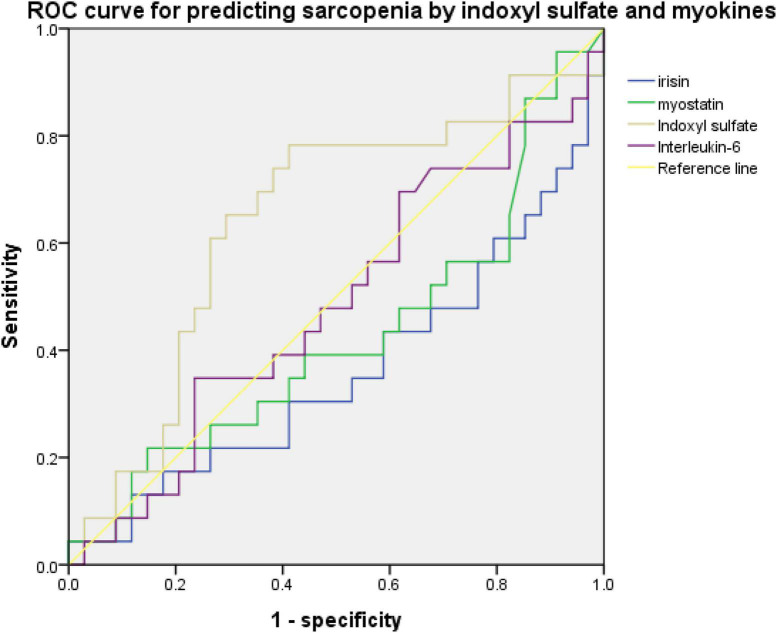
ROC curve for predicting sarcopenia based on concentrations of indoxyl sulfate, irisin, myostatin, and Interleukin-6 (indoxyl sulfate: AUC: 0.642, 95% CI: 0.489–0.795, *p* = 0.071; irisin: AUC: 0.361, 95% CI: 0.208–0.513, *p* = 0.076; myostatin: AUC: 0.426, 95% CI: 0.269–0.0.583, *p* = 0.345; interleukin-6: AUC: 0.578, 95% CI: 0.429–0.727, *p* = 0.317).

**FIGURE 3:**
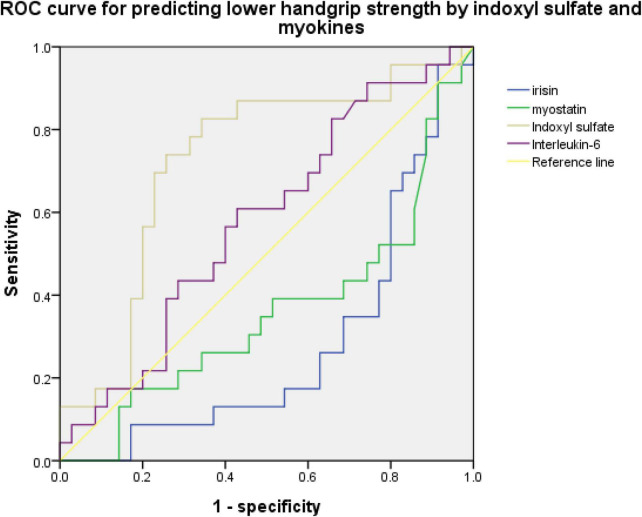
ROC curve for predicting low handgrip strength based on concentrations of indoxyl sulfate, irisin, myostatin, and interleukin-6 (indoxyl sulfate: AUC: 0.724, 95% CI: 0.585–0.863, *p* < 0.05; irisin: AUC: 0.276, 95% CI: 0.142–0.410, *p* < 0.05; myostatin: AUC: 0.358, 95% CI: 0.208–0.508, *p* < 0.05; interleukin-6: AUC: 0.578, 95% CI: 0.429–0.727, *p* = 0.317).

An ROC curve for predicting sarcopenia or low handgrip strength was used to investigate the relationship between myokine and indoxyl sulfate concentrations. Figures 2, 3 present the ROC curves for predicting sarcopenia and low handgrip strength based on myokine and indoxyl sulfate in the total population. Figure 2 illustrates the ROC curve for predicting sarcopenia. On the basis of this model, the concentrations of indoxyl sulfate, irisin, myostatin, and interleukin-6 were not predictive of sarcopenia (indoxyl sulfate: AUC: 0.642, 95% CI: 0.489–0.795, *p* = 0.071; irisin: AUC: 0.361, 95% CI: 0.208–0.513, *p* = 0.076; myostatin: AUC: 0.426, 95% CI: 0.269–0.0.583, *p* = 0.345; interleukin-6: AUC: 0.578, 95% CI: 0.429–0.727, *p* = 0.317). Figure 3 illustrates the ROC curve for predicting low handgrip strength. The concentrations of indoxyl sulfate and irisin were predictive of low handgrip strength in the total population (indoxyl sulfate: AUC: 0.724, 95% CI: 0.585–0.863, *p* < 0.05; irisin: AUC: 0.276, 95% CI: 0.142–0.410, *p* < 0.05). The concentrations of myostatin and interleukin-6 were not predictive of low handgrip strength (myostatin: AUC: 0.358, 95% CI: 0.208–0.508, *p* < 0.05; interleukin-6: AUC: 0.578, 95% CI: 0.429–0.727, *p* = 0.317). The cutoff values for irisin and indoxyl sulfate for diagnosing low handgrip strength were 63 pg/ml and 136 μM, respectively.

Table 5 illustrates the univariate and multivariate analyses of the factors associated with low handgrip strength. A univariate analysis was performed to investigate the odds ratios (ORs) for low handgrip strength among groups when stratified by demographic, hematological, and biochemical parameters (demographic: age; history of diabetes mellitus, congestive heart failure and hypertension; biochemical and hematological parameters: serum albumin > 3.5 g/dL, phosphorus > 5.5 mg/dL, hemoglobin > 12 g/dL). Irisin concentrations were also included in this analysis due to the association with low handgrip strength. In the univariate analysis, older age (> 65 years), higher indoxyl sulfate concentrations (> 136 μM), and history of congestive heart failure were associated with low handgrip strength [OR for indoxyl sulfate: 3.485, 95% confidence interval (CI): 1.372–8.852, *p* = 0.02; OR for age > 65 years: 3.136, 95% CI: 1.221–8.058, *p* = 0.005; OR for congestive heart failure: 1.494, 95% CI: 1.058–2.109, *p* = 0.009]. Higher irisin levels (> 63 pg/ml) were negatively correlated with low handgrip strength (OR: 0.462, 95% CI: 0.235–0.911, *p* = 0.019). In the multivariate regression analysis with age, irisin, indoxyl sulfate, congestive heart failure, higher indoxyl sulfate concentrations, and advanced age were associated with the risk of low handgrip strength (OR for age: 6.728, 95% CI: 1.418–31.33, *p* = 0.016; OR for indoxyl sulfate: 8.525, 95% CI: 1.807–40.207, *p* = 0.007). To evaluate the effect of age on the expression of myokine and handgrip strength, we divided the subjects into quartiles based on the age concentration (Table 6). The handgrip strength was the highest in the 1st quartile,(31.79 ± 8.64 kg, vs. 4th quartile with 19.23 ± 9.44 kg, *p* < 0.05) The irisin was also the highest in the 1st quartile (122.39 ± 124.96 pg/ml, vs. 4th quartile with 63.33 ± 21.85 pg/ml, *p* < 0.05).

**TABLE 5:** Univariate and Multivariate logistic regression analyses for the factors associated with low handgrip strength.

	Univariate odd ratio (95% CI)	*p*-value	Multivariate odd ratio (95% CI)	*p*-value
Age (> 65 year/old)	3.136 (1.221–8.058)	0.005	6.728 (1.418–31.33)	0.016
Without CKD (estimated GFR > 60 ml/min)	0.890 (0.643–1.232)	0.356		
Hyperphosphatemia (> 5.5 mg/L)	0.654 (0.35–1.22)	0.171		
Albumin (> 3.5 g/dL)	0.563 (0.29–1.90)	0.231		
Hemoglobin (< 12 g/dL)	0.593 (0.299–1.173)	0.097		
Irisin (> 63 pg/ml)	0.462 (0.235–0.911)	0.019		
Indoxyl sulfate (> 135 μM)	3.485 (1.372–8.852)	0.002	8.525 (1.807–40.207)	0.007
DM	0.725 (0.424–1.344)	0.25		
Hypertension	0.744 (0.546–1.098)	0.135		
Congestive heart failure	1.494 (1.058–2.109)	0.009		

**TABLE 6:** The concentration of myokine and handgrip strength between the participants based on the quartile divided by age.

Groups	1st quartile based on age (30–46 years old)	2nd quartile based on age (46–61 years old)	3rd quartile based on age (61–69 years old)	4th quartile based on age (69–85 years old)	*P*-value
Handgrip strength (kg)*	31.79 ± 8.64	25.96 ± 8.28	26.83 ± 11.19	19.23 ± 9.44	*P* < 0.05
Irisin (pg/ml)*	122.39 ± 124.96	85.14 ± 61.88	61.66 ± 27.93	63.33 ± 21.85	*P* < 0.05
Myostatin (ng/ml)	2.98 ± 5.07	1.59 ± 1.71	0.96 ± 0.47	1.05 ± 0.53	*P* = 0.087
Interleukin 6 (pg/ml)	3.40 ± 3.00	4.66 ± 6.05	3.54 ± 4.07	4.44 ± 7.19	*P* = 0.634

*1st quartile vs. 4th quartile.

**FIGURE 4:**
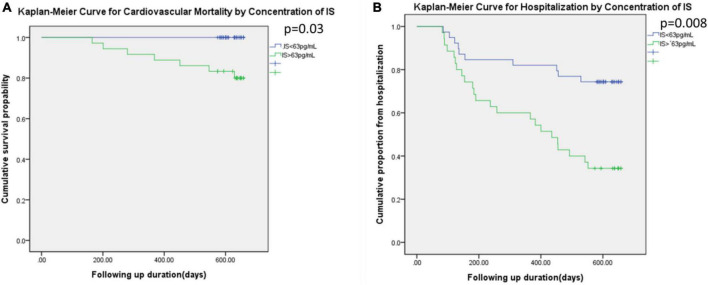
Kaplan–Meier curve for the cardiovascular mortality **(A)** and the hospitalization **(B)** based on the IS level (higher than 63 pg/ml or not).

Figure 4 illustrated the Kaplan–Meier curve for cardiovascular mortality and the hospitalization based on the higher or lower concentration of indoxyl sulfate. The concentration of participants with higher indoxyl sulfate concentration (> 63 pg/ml) was associated with higher incidence of cardiovascular mortality (*p* = 0.03) and higher hospitalization rate (*p* = 0.008) under duration of 600 days.

### 3.4. Indoxyl sulfate was associated with decreased gynoid total mass

Table 7 displays the DEXA parameters of the participants in all three groups. The appendicular skeletal muscle indices were similar among the groups (6.89 ± 1.32, 7.14 ± 0.90, and 6.82 ± 1.20 kg/m^2^ for the control, CKD, and ESRD groups, respectively; *p* = 0.641). The control group had the lowest android to gynoid and trunk to limb fat mass ratios among the three groups (android/gynoid: 1.11 ± 0.31, 1.40 ± 0.30, 1.21 ± 0.18, *p* < 0.05; trunk/limb: 1.22 ± 0.29, 1.55 ± 0.46, 1.56 ± 0.26, *p* < 0.05 for the control, CKD, and ESRD groups, respectively). Table 8 reveals the correlations between DEXA parameters and indoxyl sulfate concentrations in the total population. The gynoid total mass was negatively correlated with indoxyl sulfate concentration (*r* = −0.304, *p* = 0.016). Table 9 reveals the difference in gynoid parameters between patients with and without low handgrip strength in the total population. The total and lean gynoid mass were lower in participants with low handgrip strength. Table 10 illustrated the correlation between indoxyl sulfate concentration with other hematologic or biochemical parameters with difference between groups in Table 3. The concentration of indoxyl sulfate was positive correlated with the concentration with phosphorus (*r* = 0.363, *p* < 0.05). There was no other correlation between indoxyl sulfate and other parameters.

**TABLE 7:** Dual-energy X-ray absorptiometry parameters by group.

	Control	CKD	ESRD	*p*-value
Android fat mass (kg)	2.16 ± 0.87	2.11 ± 0.95	2.09 ± 0.80	0.969
Android lean mass (kg)	3.64 ± 0.67	3.79 ± 1.06	3.63 ± 0.86	0.814
Android total mass (kg)	5.08 ± 1.38	5.97 ± 1.71	5.73 ± 1.52	0.870
Android fat percentage (%)	36.34 ± 7.08	35.45 ± 9.01	36.04 ± 6.45	0.944
Gynoid fat mass (kg)	3.50 ± 1.32	2.82 ± 1.00	2.75 ± 0.85	0.081
Gynoid lean mass (kg)	7.26 ± 1.20	7.03 ± 1.95	6.51 ± 1.25	0.242
Gynoid total mass (kg)	10.85 ± 1.81	9.89 ± 2.42	9.26 ± 1.73	0.059
Gynoid fat percentage (%)	32.00 ± 8.78	28.40 ± 7.79	29.60 ± 6.05	0.407
Android/gynoid fat ratio	1.11 ± 0.31	1.40 ± 0.30	1.21 ± 0.18	<0.05
The ratio of trunk/leg fat mass	1.12 ± 0.26	1.40 ± 0.47	1.28 ± 0.20	0.05
The ratio of total trunk/limb mass	1.22 ± 0.29	1.55 ± 0.46	1.56 ± 0.26	<0.05
Lean mass/height^2^	16.64 ± 2.65	16.78 ± 1.60	16.86 ± 2.24	0.949
Appendicular skeletal muscle/height^2^	6.89 ± 1.32	7.14 ± 0.90	6.82 ± 1.20	0.641

**TABLE 8:** Correlations between indoxyl sulfate and body composition in total population.

	Indoxyl sulfate	*p*-value
Android fat mass (kg)	-0.029	0.823
Android lean mass (kg)	-0.004	0.974
Android total mass (kg)	-0.025	0.846
Android fat percentage (%)	-0.24	0.852
Gynoid fat mass (kg)	-0.248	0.052
Gynoid lean mass (kg)	-0.227	0.076
Gynoid total mass (kg)	-0.304	0.016
Gynoid fat percentage (%)	-0.092	0.47
Android/gynoid fat ratio	0.024	0.849
The ratio of trunk/leg fat mass	0.077	0.586
The ratio of total trunk/limb mass	0.211	0.089
Lean mass/height^2^	0.183	0.141
Appendicular skeletal muscle/height^2^	0.19	0.133
Fat mass/height^2^	-0.215	0.083

**TABLE 9:** Comparison of gynoid total mass, lean mass, and fat mass in participants with and without low handgrip strength.

	With low handgrip strength	Without low handgrip strength	*p*-value
Gynoid fat mass (kg)	2.61 ± 0.63	3.08 ± 1.22	0.097
Gynoid lean mass (kg)	6.40 ± 1.38	7.30 ± 1.40	0.039
Gynoid total mass (kg)	9.02 ± 1.68	10.42 ± 1.97	0.017
Gynoid fat percentage (%)	29.20 ± 5.71	29.14 ± 8.65	0.98

**TABLE 10:** The correlation between the concentration of indoxyl sulfate and age, hematologic or biochemical parameters with difference between groups in Table 3.

	Correlation coefficient	*p*-value
Age	-0.179	0.263
Sodium	-0.260	0.101
Phosphorus	0.363	0.018
Albumin	0.001	0.996
Hemoglobin	-0.130	0.419

## 4. Discussion

In our study, low handgrip strength was common among patients with CKD and ESRD. We observed a correlation between low handgrip strength and high hospitalization rates, but no such association existed between sarcopenia and hospitalization. Indoxyl sulfate concentration was an important factor contributing to low handgrip strength, and the multivariate logistic regression analysis revealed that indoxyl sulfate counteracted the protective effect of irisin. Furthermore, the indoxyl sulfate concentration was inversely correlated with gynoid total mass.

The DEXA scan utilized two low-dose X-ray beams of different levels to detect fat, bone mass, and muscle mass. The results revealed decreased appendicular skeletal muscle mass relative to height (kg/m^2^), and the percentages of appendicular skeletal muscle wasting were similar among the groups. Two diagnostic criteria are used for identifying sarcopenia in patients receiving peritoneal dialysis or hemodialysis: (1) an appendicular skeletal muscle index more than two standard deviations below the reference index for healthy young adults (26–28) and (2) an appendicular lean mass index at least 20 percentiles below the general population (28). The incidence of sarcopenia varies from 20 to 73.5% depending on the diagnostic criteria. DEXA serves as an important tool for diagnosing sarcopenia, but several factors can impair the diagnostic efficacy in patients with CKD. Nevertheless, variations in fat, bone mass, and muscle mass were noted in the DEXA results. In non-dialysis CKD, visceral adipose tissue is highly associated with obesity, and adipose tissue is associated with metabolic syndrome or cardiovascular complications such as coronary artery calcification (29, 30). In patients undergoing dialysis, hydration status and the modality of renal replacement therapy influence the results of body composition scans. In patients receiving peritoneal dialysis, the amount of peritoneal dialysate might influence the patient’s hydration status and result in an overestimation of muscle mass (31). Therefore, sarcopenia diagnoses can be inconsistent across study groups. Our study demonstrated that low handgrip strength was a more reliable predictor of hospitalization and poor clinical outcomes during the 2-year follow-up than radiologically diagnosed sarcopenia, as Figure 1 illustrated. Low handgrip strength was associated with higher mortality and the development of chronic illnesses such as malignant cancer among patients in all age groups (32–34). Although the ability of handgrip strength to represent overall muscle strength has not been determined, handgrip strength can serve as an indicator of cardiovascular fitness (35–36). In CKD patients, low handgrip strength was associated with comorbidities such as hypernatremia (19) and poor renal outcomes (37). Besides, several comorbidities common in CKD/ESRD could influence the handgrip strength, such as anemia (38, 39). The accumulation of uremic toxin, such as indoxyl sulfate, could also influence overall fitness vial influencing ventricular remodeling or impairment of immunity (40, 41). Therefore the concentration of indoxyl sulfate also reflected the 2-year cardiovascular mortality and the hospitalization in our study (Figure 4). The overall fitness, indicated by lower handgrip strength, was associated with the higher hospitalization by infection as previous reports (42, 43). Therefore, the overall decreased fitness, rather than the severe decrease in skeletal mass by DEXA parameters, was more associated the clinical outcome in vulnerable subjects, such as CKD patients.

Indoxyl sulfate is a protein-bound uremic toxin derived from tryptophan. After tryptophan is ingested, it is transformed into indole derivatives and indoxyl sulfate by the hepatic cytochrome P450 2E1. The organic anion transporter (OAT) within the renal proximal tubules normally governs the excretion of indoxyl sulfate, but in patients with CKD, tubulointerstitial fibrosis decreases OAT function and leads to increased plasma concentrations of indoxyl sulfate (16, 4). Indoxyl sulfate causes cellular damage either through direct generation of oxidative stress or by modulating transcription factors such as aryl hydrocarbon receptors (44). In patients receiving maintenance hemodialysis or peritoneal dialysis (45, 46), indoxyl sulfate could not be removed effectively, and the toxic effect of indoxyl sulfate on skeletal muscle has been demonstrated *in vivo* and *in vitro*. As the Figure 4 illustrated, the higher concentration of indoxyl sulfate was associated higher cardiovascular mortality and hospitalization in total population. Based on the previous study, the indoxyl sulfate might influence the handgrip strength vial: (1) the decrease ATP generation in mitochondria by inducing the endoplasmic reticulum stress in skeletal by dysregulating tricarboxylic acid cycle (17); (2) inducing the myogenesis by arousing the unfolded protein response in endoplasmic reticulum (6); (3) influencing the overall fitness vial the decrease cardiac function, impairment of the hematopoiesis, or angiogenesis of peripheral arterys (47, 40, 48). We noticed that the indoxyl sulfate concentration was negatively associated with total gynoid mass. Additionally, low handgrip strength was correlated with low gynoid total and lean mass. This finding corresponds to the findings of Chao et al. who reported that lower total body mass and lean mass were associated with frailty in patients undergoing dialysis. Chao et al. also noted that appendix fat, in contrast with trunk fat, was higher in patients with frailty (49). A decrease in weight-bearing capacity might further impair the cardiovascular fitness of patients with CKD and ESRD (50). In light of our results, we believe that protein-bound uremic toxins such as indoxyl sulfate might influence the weight-bearing lean skeletal muscle mass of the gynoid area. However, further research into the pathophysiological mechanisms is needed to verify this theory.

Irisin is the myokine released from skeletal muscle during exercise. Irisin expression is regulated by the peroxisome proliferator-activated receptor γ (PPARγ) coactivator 1α (PGC-1α) and fibronectin type III domain containing 5 (FNDC5) axis (51). After being released from skeletal muscle, irisin modulates the energy expenditure in adipose tissue by stimulating the expression of brown preadipose genes in beige precursor fat cells (52). Additionally, irisin could activate osteoblast differentiation (53) or inhibit the apoptosis of osteoblasts by reducing the generation of inflammasomes (54). Irisin also maintains osteocytic survival by binding to the αV class of integrins (55). Furthermore, irisin could modulate the expression of mitochondrial uncoupling protein 2 to reduce oxidative stress after ischemia or reperfusion injury. Because obesity and metabolic syndrome develop before ESRD, irisin could counteract the hazard of obesity and therefore provide renoprotection (56). Serum concentrations of irisin decrease in patients with CKD. Wen et al. demonstrated that serum irisin concentration was negatively associated with the serum concentrations of blood urea nitrogen and creatinine (57, 58). A lower concentration of irisin was associated with cardiovascular mortality in patients with CKD (59). Our study is the first to demonstrate that irisin concentration is negatively correlated with lower muscle strength in patients with CKD without influence on lean muscle mass composition. As mentioned, protein-bound uremic toxins such as indoxyl sulfate might hamper mitochondrial ATP generation, counteracting the protective effect of irisin in several aspects. Patients with CKD have several risk factors that dysregulate the PGC-1α–FNDC5 axis, such as vitamin D deficiency and the accumulation of protein-bound uremic toxins (60, 61). An *in vitro* study demonstrated that indoxyl sulfate dysregulated the expression of PPARγ in C2C12 cells and therefore increased autophagy levels (62). On the basis of this evidence, we believe that indoxyl sulfate might counteract the effect of irisin and contributed to the lower muscle strength observed in patients with CKD and ESRD. Nevertheless, further investigation is needed to clarify the detailed mechanisms of irisin expression and to explore possible interventions for patients with low handgrips, such as vitamin D supplements or exercise (63, 64). In contrast to the situation regarding irisin, we observed no relationship between myostatin alone and low handgrip strength in this cohort study, and the concentration of myostatin was even higher in control group (without statistic difference). Myostatin, as part of the TGF-beta superfamily, is secreted from skeletal muscle and serves as the negative regulator of myocytes. The active form of myostatin inhibits the phosphoinositide 3-kinase–protein kinase B signaling pathway of skeletal muscle (65) and induces apoptosis of myocytes through regulation of gene-expressed autophagy. Notably, apoptosis and myostatin mRNA are upregulated in the skeletal muscle of patients with CKD (66). A possible mechanism is that the percentage of congestive heart failure is higher in CKD and ESRD group. It has been noticed that the myostatin serves as the inhibitor to alleviate the development of the cardiac fibrosis, and the origin of myostatin might be derived from the cardiac and adipose tissue (67, 68). Therefore, the influence of myostatin on sarcopenia was equivocal in the patients with CKD and ESRD (69, 70). We also observed that the concentration of interleukin-6 was lower in the control group without statistic difference. Interleukin-6 has been linked to lower handgrip strength in other studies because the concentration reflected the inflammatory status along with the aging process (71, 72). The mean value was higher in the subjects with higher handgrip strength without statistic difference. A possible explanation was the difference in gender. Miko et al. demonstrated that the higher plasma IL-6 concentration in male gender was associated with better skeletal muscle condition (73). The female percentage was higher in the control group, and therefore the IL-6 concentration might be lower in the control group. The interactions of anabolic or catabolic myokines with the regulators for myokines in patients with CKD warrant further advanced study. On the basis of our results, we believe the protective effect of irisin might be mitigated by factors other than myokines.

Hypoalbuminemia has been noticed as a possible factor contributing to lower handgrip strength in our study. Hypoalbuminemia and other indicators for malnutrition are associated with cardiovascular events in CKD patients (74). For the CKD patients with or without dialysis, the dietary restriction of protein and sodium might decrease the adequate calorie uptake and therefore worsen the malnutrition (75, 76). Beyond the insufficient calorie intake, the underlying factors such as insulin resistance, vitamin D deficiency or excessive homocysteine (77, 78) could worsen the inflammatory status in CKD patients and therefore worsen the cardiovascular function. The assessment for screening the malnutrition-inflammation-atherosclerosis syndrome (MIA syndrome), the conventional Subjective Global Assessment or protein energy wasting are important for the routine care in CKD patients (79, 80). Besides, the measurement for inflammatory indicators such as C-reactive protein might be important for caring the CKD patients (81, 82). From our study, the body mass index was similar between groups. Therefore the indicators for inflammation, which were not measured in our study, and its interaction with uremic toxin or myokine might be helpful for providing the possible therapeutic strategies in managing sarcopenia/frailty in CKD patients.

This study has several limitations. First, the study was initiated in the single institute. Second, the influence of age for the lower handgrip strength was noted in our study from the multivariate logistic regression (odd ratio: 6.728, 95% confidence interval: 1.418–31.33). Our data also demonstrated that the irisin concentration was lower in the elder subjects (83). It has been noticed that the age could influence the handgrip strength, although the factors contributing for lower handgrip strength variated in different studies with different designs (84–86). The influence of aging might also be altered by increasing the sample size or altering the enrollment criteria accordingly. Third, the measurement for indoxyl sulfate in the study was the ELISA method. The detection of indoxyl sulfate is mostly by the liquid chromatography - mass spectrometry (LC–MS) and high–performance liquid chromatography (HPLC) (87, 88). Although the validation has been validated, to measure the concentration of indoxyl sulfate with LC-MS or HPLC might provide more direct evidence between the protein bounded uremic toxin and clinical events. Fourth, according to the literature, handgrip strength, in contrast with lean muscle mass, predicts clinical outcomes. Our study revealed a cross-sectional, but not longitudinal, correlation between uremic toxins and myokines. A longitudinal follow-up might provide a better understanding of the variation in loss of handgrip strength. On the basis of other studies, we speculate that the administration of recombinant irisin might improve the muscle expression of irisin and therefore influence skeletal muscle mass (89). However, we still lack a specific therapeutic strategy for maintaining grip strength as opposed to skeletal muscle mass. Further therapeutic strategies are necessary for promoting the maintenance of muscle strength; therefore, longitudinal studies and *in vivo* studies should be initiated to research the interaction between uremic toxins and the expression of myokines in patients with CKD.

In conclusion, low handgrip strength—but not skeletal muscle mass—was associated with hospitalization in patients with CKD and ESRD. Low handgrip strength was also associated with higher serum concentrations of uremic toxins (namely, indoxyl sulfate) and lower concentrations of irisin compared with the general population.

## Data availability statement

The original contributions presented in this study are included in the article/supplementary material, further inquiries can be directed to the corresponding author.

## Ethics statement

The studies involving human participants were reviewed and approved by the Cardinal Tien Hospital CTH-107-3-5-027. The patients/participants provided their written informed consent to participate in this study.

## Author contributions

K-CL: conceptualization, methodology, and software. S-ML: data curation and writing—original draft preparation. C-MZ and C-LL: visualization and investigation. W-CL and K-CH: supervision. Y-ML and M-TL: software and validation. Y-CH: writing—reviewing and editing. All authors contributed to the article and approved the submitted version.
